# *Burkholderia cenocepacia* and *Salmonella enterica* ArnT proteins that transfer 4-amino-4-*deoxy*-l-arabinose to lipopolysaccharide share membrane topology and functional amino acids

**DOI:** 10.1038/srep10773

**Published:** 2015-06-01

**Authors:** Faviola Tavares-Carreón, Kinnari B. Patel, Miguel A. Valvano

**Affiliations:** 1 Centre for Human Immunology, Department of Microbiology and Immunology, University of Western Ontario, London, Ontario, N6A 5C1, Canada; 2 Centre for Infection and Immunity, Queen’s University Belfast, Belfast, BT9 7AE, United Kingdom

## Abstract

We recently demonstrated that incorporation of 4-amino-4-*deoxy*-l-arabinose (l-Ara4N) to the lipid A moiety of lipopolysaccharide (LPS) is required for transport of LPS to the outer membrane and viability of the Gram-negative bacterium *Burkholderia cenocepacia*. ArnT is a membrane protein catalyzing the transfer of l-Ara4N to the LPS molecule at the periplasmic face of the inner membrane, but its topology and mechanism of action are not well characterized. Here, we elucidate the topology of ArnT and identify key amino acids that likely contribute to its enzymatic function. PEGylation assays using a cysteineless version of ArnT support a model of 13 transmembrane helices and a large C-terminal region exposed to the periplasm. The same topological configuration is proposed for the *Salmonella enterica* serovar Typhimurium ArnT. Four highly conserved periplasmic residues in *B. cenocepacia* ArnT, tyrosine-43, lysine-69, arginine-254 and glutamic acid-493, were required for activity. Tyrosine-43 and lysine-69 span two highly conserved motifs, ^42^RYA^44^ and ^66^YFEKP^70^, that are found in ArnT homologues from other species. The same residues in *S. enterica* ArnT are also needed for function. We propose these aromatic and charged amino acids participate in either undecaprenyl phosphate-l-Ara4N substrate recognition or transfer of l-Ara4N to the LPS.

The Gram-negative opportunistic bacterium *Burkholderia cenocepacia* causes serious lung infections in patients with cystic fibrosis^1^,^2^. *B. cenocepacia* is difficult to treat because of its extraordinarily high intrinsic resistance to virtually all clinically useful antibiotics including antimicrobial peptides such as the polymyxins^3^,^4^,^5^. Intrinsic resistance of *B. cenocepacia* depends in part on its lipopolysaccharide (LPS)^6^,^7^,^8^. *B. cenocepacia* can modify its LPS lipid A moiety by incorporating 4-amino-4-*deoxy*-l-arabinose (l-Ara4N). The LPS inner core oligosaccharide contains a side branch d*-*glycero-d-*talo*-octo*-*2*-*ulosonic acid, which is also modified by l-Ara4N^9^,^10^. These modifications neutralize negative charges in the LPS molecule, inhibiting binding of cationic molecules^11^. Remarkably, l-Ara4N modification in *B. cenocepacia* is essential for bacterial viability^12^, since it is required for LPS export to the outer membrane^13^, and for high resistance to polymyxin B (PmB) at concentrations reaching milligram range^4^,^7^,^8^,^13^,^14^.

l-Ara4N biosynthesis occurs in the cytoplasm by the enzymes AraABCD. This process converts UDP-glucuronic acid into l-Ara4N linked to undecaprenyl phosphate^15^. Undecaprenyl phosphate-l-Ara4N is translocated across the inner membrane by the heterodimer ArnE-ArnF flippase^16^ and finally ArnT transfers l-Ara4N to the lipid A in a reaction occurring at the periplasmic face of the inner membrane^17^. Hamad *et al.*^13^ postulated that *B. cenocepacia* ArnT also transfers l-Ara4N to the inner core d*-*glycero-d-talo*-octo-2-*ulopyranosoic acid, but this was not directly demonstrated. Because the transfer of l-Ara4N to the LPS is essential for *B. cenocepacia* viability^12^,^13^, ∆*arnT* mutants are only viable if a suppressor mutation occurs in the *lptG* gene encoding a component of the LPS transport pathway, which allows export of unmodified LPS^13^.

Lipid A modifications requiring ArnT family proteins have been reported in *Escherichia coli*, *Salmonella enterica* serovar Typhimurium and *Pseudomonas aeruginosa* where LPS is modified with l-Ara4N^18^,^19^, and in *Francisella novicida* and two *Bordetella* species *(B. bronchiseptica and B. pertussis)* where LPS is modified with galactosamine and glucosamine, respectively^20^,^21^,^22^. Since LPS modifications contribute to pathogenicity and innate immunity evasion, ArnT can be considered a virulence factor in these bacteria.

ArnT is an integral membrane protein; its topology was partially characterized in *S. enterica*^23^, but little is known on functional regions and enzyme mechanism. Elucidating the topology of ArnT is critical to understand its function and developing molecules targeting l-Ara4N modification of LPS, which would abolish intrinsic bacterial resistance to antimicrobial peptides. In this study, we characterize the topology of *B. cenocepacia* and *S. enterica* ArnT. Both proteins show very similar topology, suggesting the topological arrangement in other proteins of the ArnT family is likely conserved. We propose ArnT proteins consist of thirteen transmembrane domains with two large periplasmic loops and a C-terminal segment that is exposed to the periplasm. We also identify four highly conserved periplasmic amino acids that are presumably involved in ArnT function.

## Results

### ArnT is a membrane protein with 13 predicted transmembrane helices

To facilitate immunodetection while exploring the ArnT topology we constructed a recombinant protein carrying a C-terminal FLAG-10xHis tag. ArnT_FLAG-10xHis_ was expressed under the control of a rhamnose-inducible promoter in the ∆*arnT-arnBC*^*+*^*lptG*_D31H_ suppressor strain (herein ∆*arnT*)^13^. This *B. cenocepacia* strain is viable but highly sensitive to PmB due to lack of l-Ara4N in the LPS^13^. In the presence of rhamnose, ArnT_FLAG-10xHis_ restored ∆*arnT* PmB resistance to parental levels (Supplementary Figure S1A online), and was detected by immunoblot as a polypeptide with an apparent mass of 63 kDa, in agreement with its predicted mass of 66.3 kDa (Supplementary Figure S1B online). These experiments demonstrate that the FLAG-10xHis tag does not affect ArnT function and membrane localization.

**Figure 1:**
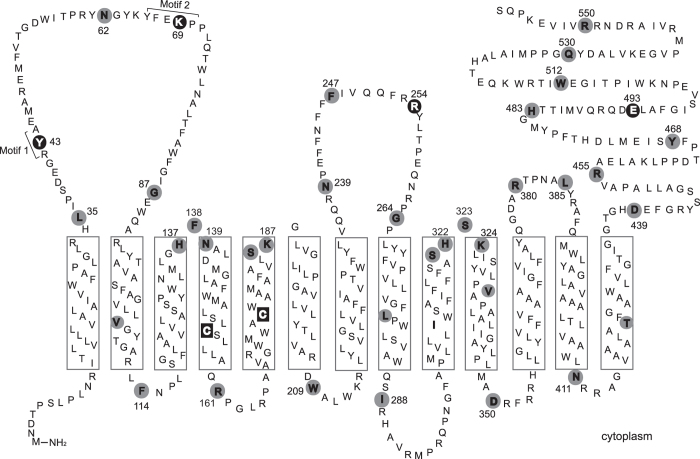
ArnT topological model. The model was derived using PolyPhobius and displayed with TMRPres2D. The ArnT C-terminus is oriented towards the periplasm and the protein has 13 predicted transmembrane helices, indicated by rectangles. The residues replaced by cysteine are shown in gray circles. Functional residues are indicated by black circle. The endogenous cysteines of ArnT are shown in black squares.

**Figure 2:**
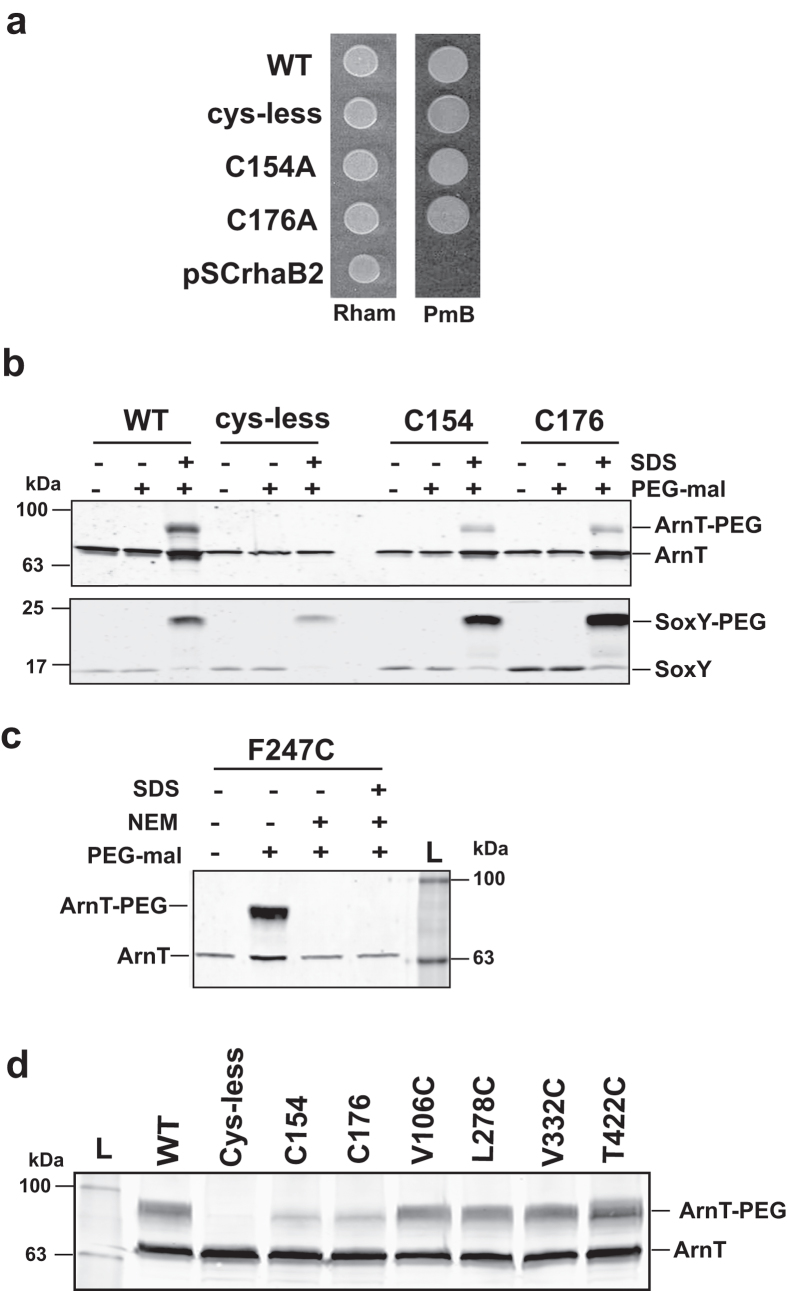
Native cysteines in ArnT are located in transmembrane segments. (**a**) Cys-less ArnT protein remains functional. Complementation of the ∆*arnT-arnBC*^*+*^ suppressor strain transformed with pSCrhaB2 encoding ArnT_Cys-less_, ArnT_C154A_, and ArnT_C176A_. Cells expressing ArnT_FLAG-10xHis_ and the vector pSCrhaB2 were used as controls. The transformants were spotted on LB supplemented with 0.4% of l-rhamnose (Rham) and 10 μg ml^−1^ PmB. (**b**) *E. coli* DH5α containing a plasmid encoding SoxY was transformed with plasmids expressing ArnT_Cys-less_, ArnT_C154A_ and ArnT_C176A_. Bacteria were grown to mid-exponential phase and protein expression induced with 0.2% l-arabinose. Cells were harvest and resuspend in 0.3 ml of HEPES/MgCl_2_ buffer. 0.1 ml of cell suspension was incubated with buffer alone or 1 mM PEG-mal with or without 2% SDS for 1h at room temperature. Reactions were quenched with 45 mM DTT, and proteins were separated by SDS-PAGE and transferred to nitrocellulose membrane. ArnT derivatives were detected with anti-FLAG and SoxY with anti-hemagglutinin serum. (**c**) For NEM blocking, cells were harvested and resuspended in 0.4 ml of HEPES/MgCl_2_ buffer, then were incubated with 5 mM NEM for 30 min to block accessible sulfhydryl groups. The pellet was washed twice with 0.5 ml of ice-cold HEPES/MgCl_2_ buffer and finally was PEGylated as indicated for panel b. (**d**) Cells were harvested and resuspended in 0.4 ml of HEPES/MgCl_2_ buffer. 0.1 ml of the cell suspension was incubated with 5 mM NEM for 30 min, and then was treated with 1 mM PEG-mal and 2% SDS as indicated in panel *B*. The position of the molecular mass marker is indicated to the left, and the position of PEGylated and non-PEGylated protein to the right.

Earlier work suggested that *S. enterica* and *E. coli* ArnT proteins contain 12 predicted transmembrane helices and that the C-terminus is in the cytosolic compartment^17^,^23^. However, an *in silico* model of *B. cenocepacia* ArnT topology with PolyPhobius predicted 13 transmembrane helices and a large C-terminus exposed to the periplasmic space in both *S. enterica* and *E. coli* ArnT proteins (Fig. 1). To provide experimental evidence for the predicted ArnT transmembrane topology we used the substituted cysteine accessibility method with methoxypolyethylene glycol maleimide (PEG-mal) labeling^24^,^25^. PEG-mal is a maleimide conjugate that modifies cysteines but cannot cross membranes. Reaction of a cysteine with PEG-mal results in a mass addition of ~5 kDa to the protein, which appears as a band shift by immunoblotting. PEG-mal modification using EDTA-permeabilized whole cells indicates that the cysteine has a periplasmic orientation, while PEG-mal modification under denaturing conditions (presence of SDS) suggests that the cysteine is within a transmembrane helix or faces the cytoplasm. *B. cenocepacia* ArnT_FLAG-10xHis_ has two cysteine residues at positions 154 and 176, which were replaced by alanine to generate a cysteineless derivative (ArnT_Cysless_). ArnT_Cysless_ and the single cysteine replacement derivatives (ArnT_C154A_ and ArnT_C176A_) restored PmB resistance in ∆*arnT* at similar levels as the wild type strain (Fig. 2A), demonstrating that the native cysteines are dispensable for ArnT function. Using PEG-mal labeling, we observed that ArnT_C154A_ and ArnT_C176A_ were only PEGylated in detergent (2% SDS), while ArnT_Cysless_ was not PEGylated under any condition (Fig. 2B). These results suggest that Cys-154 and Cys-C176 are buried in transmembrane helices, as predicted by the *in silico* model (Fig. 1). Cell membrane integrity in these experiments was confirmed by co-expressing ArnT_C154_ and ArnT_C176_ with HA-SoxY, a cytoplasmic protein that is highly PEGylated^24^. Immunoblotting using anti-HA antibodies shows that HA-SoxY was only PEGylated under denaturing conditions (Fig. 2B), demonstrating that the bacterial membranes remained intact.

To confirm whether Cys-154 and Cys-176 are indeed buried, PEGylation was performed in the presence or absence of N-ethylmaleimide (NEM). NEM is a small sulfhydryl-reactive molecule that crosses membranes, but cannot react with cysteines embedded within the membrane bilayer^26^. As a control, we used a phenylalanine-247 cysteine replacement mutant, since this residue is predicted to reside in the periplasmic side. PEGylation of the resulting protein was examined in samples pretreated with or without NEM, and also with or without SDS. This experiment showed that ArnT_F247C_ could be PEGylated in the absence of NEM (Fig. 2C), demonstrating a periplasmic orientation for this residue. In contrast, ArnT_C154A_ and ArnT_C176A_ were PEGylated despite treatment with NEM, demonstrating that the single cysteine remaining in each protein (Cys-176 in ArnT_C154A_ and Cys-154 in ArnT_C176A_) is inaccessible to NEM modification (Fig. 2D), as would be expected if these residues are buried in the membrane bilayer. These results support the conclusion that the native cysteine residues in ArnT are in a transmembrane helix, as predicted by the *in silico* topological model.

More experimental evidence for the predicted ArnT topology was obtained by introducing 38 novel cysteine replacements at various positions of ArnT_Cysless_. As before, mutant proteins were analyzed by immunoblotting to confirm that the cysteine substitution did not affect protein stability and membrane location (Supplementary Figure S2 online). Also, the ArnT cysteine replacements restored PmB resistance in ∆*arnT* to wild type levels. We then analyzed the topology of amino acids predicted to reside in transmembrane segments. Cysteine residues replacing valine-106, leucine-278, valine-332, and threonine-422 resulted in ArnT proteins that could only be PEGylated in SDS irrespective of NEM pretreatment (Fig. 2D), indicating that these amino acids are embedded in the membrane bilayer, as predicted by the *in silico* model.

**Figure 3:**
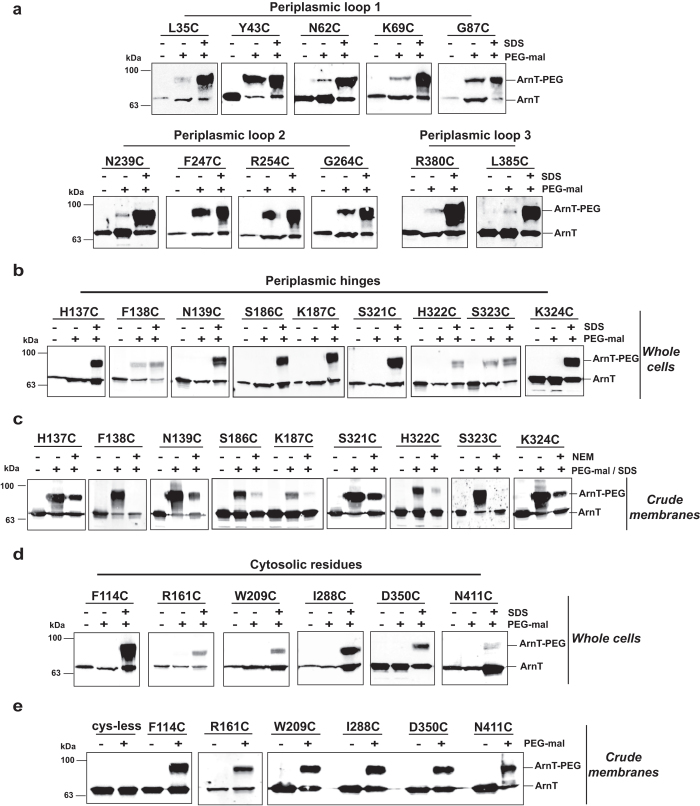
PEG-mal labeling of periplasmic and cytoplasmic ArnT amino acids. (**a**, **b**, and **d**) *E. coli* DH5α carrying plasmids encoding ArnT cysteine replacements were grown to mid-exponential phase and protein expression induced with 0.2% l-arabinose. Cells were harvested and resuspended in 0.3 ml of HEPES/MgCl_2_ buffer. 0.1 ml of cell suspension was incubated with buffer alone or 1 mM PEG-mal with or without 2% SDS for 1 h at room temperature. Reactions were quenched with 45 mM DTT, and proteins were separated by SDS-PAGE and transferred to nitrocellulose membranes, followed by immunoblot with anti-FLAG antibodies. (**c**) Crude membrane fractions were incubated with buffer alone or pre-treated with 5 mM NEM, after that membrane were incubated with 1 mM PEG-mal and 2% SDS for 1 h at room temperature. Reactions were quenched with 45 mM DTT. (**e**) Crude membrane fractions were isolated by ultracentrifugation and divided in two aliquots. One aliquot was incubated with buffer alone and the other with 1 mM PEG-mal. The reactions were quenched with 45 mM DTT and separated by SDS-PAGE. Immunoblot was probed with anti-FLAG antibody.

Next, we examined cysteine replacements in leucine-35, tyrosine-43, asparagine-62, lysine-69, glycine-87, asparagine-239, phenylalanine-247, arginine-254, glycine-264, arginine-380 and leucine-385. All of the resulting proteins could be PEGylated under non-denaturing conditions in whole cells, indicating that these residues are exposed to the periplasm (Fig. 3A). In contrast, proteins with cysteine replacements in residues located in predicted periplasmic hinges such as histidine-137, asparagine-139, serine-186, lysine-187, serine-321, histidine-322 and lysine-324, only reacted with PEG-mal upon SDS treatment (Fig. 3B), suggesting these residues are most likely embedded in the bilayer. However, ArnT_F138C_ and ArnT_S323C_ were PEGylated under non-denaturing conditions, indicating that these residues are exposed to the periplasm (Fig. 3B). To confirm this assertion we labeled total membrane preparations with and without NEM pretreatment to differentiate between solvent exposed and lipid bilayer buried residues. Membrane preparations containing proteins with cysteine replacements in histidine-137, asparagine-139, serine-186, lysine-187, serine-321, histidine-322, and lysine-324 were PEGylated in the presence of NEM, indicating that these residues are not solvent-exposed (Fig. 3C); while ArnT_F138C_ and ArnT_S323C_ only were PEGylated in the absence of NEM (Fig. 3C), demonstrating a periplasmic orientation for these residues. The intensity of the PEGylated band varied in some cases such as in ArnT_L35C_ or ArnT_F138C_ when examined by PEGylation of whole-cell preparations (Fig. 3A), and ArnT_S186C_ or ArnT_K187C_ when examined directly from membrane preparations (Fig. 3C). Because all the ArnT mutant proteins are equally expressed, we attributed reduced PEGylation to close proximity of the residue to the membrane bilayer border.

Cysteine replacements at phenylalanine-114, arginine-161, tryptophan-209, isoleucine-288, aspartic acid-350, and asparagine-411 resulted in proteins that were only PEGylated under prior denaturing conditions (Fig. 3D), suggesting they are on the cytosolic face of the membrane. These residues were also PEGylated in membrane preparations (Fig. 3E), as the conditions used for membrane disruption favor formation of inverted vesicles allowing exposure of cytoplasmic residues^27^,^28^. Collectively, the experimental results support the *in silico* predicted ArnT topology.

### The C-terminus of *B. cenocepacia* and *S. enterica* ArnT proteins resides in the periplasm

**Figure 4:**
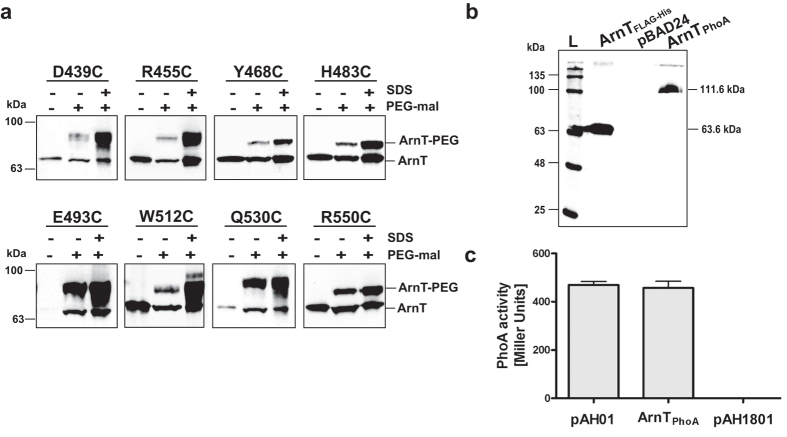
The ArnT C-terminus resides in the periplasmic space. (**a**) Whole cells from *E. coli* strain DH5α carrying plasmids encoding cysteine-substitution of the C-terminal of ArnT were treated with buffer alone or 1 mM PEG-mal with or without 2% SDS for 1 h at room temperature and quenched with 45 mM DTT. Proteins were separated by SDS-PAGE and transferred to nitrocellulose membrane for blotting with anti-FLAG to detected ArnT. (**b**) Immunoblot of total membranes from *E. coli* CC118 cells expressing _FLAG_-ArnT-_PhoA_ performed with anti-FLAG. L, BLUeye prestained protein ladder. (**c**) Quantification of PhoA activity in CC118 *E. coli* expressing _FLAG-_ArnT_PhoA_. Cells were grown in LB and induced with 0.2% arabinose. Alkaline phosphatase activity was determined by measuring the rate of PNPP hydrolysis. pAH01 encodes _FLAG_-Wzx-K367-_PhoA_ and pAH1801 encodes _FLAG_-Wzx-T242-_PhoA_, which were used as positive and negative controls, respectively^30^. The values represent the mean ± SE from three independent experiments.

According to the *in silico* topological model, *B. cenocepacia* ArnT contains an extended C-terminal segment located towards the periplasm. To investigate the orientation of the 123-amino acid soluble C-terminal region of *B. cenocepacia* ArnT, we introduced eight cysteine replacements at aspartic acid-439, arginine-455, tyrosine-468, histidine-483, glutamic acid-493, tryptophan-512, glutamine-530, and arginine-550. The corresponding proteins were PEGylated in whole cells under non-denaturing conditions, demonstrating that these residues are exposed to the periplasm (Fig. 4A). We also constructed a version of ArnT that was C-terminally fused to PhoA (_FLAG_ArnT_PhoA_), a protein probing for periplasmic location^29^, which was expressed in the *E. coli* ∆*phoA* CC118 strain. Immunoblotting of total membranes with the anti-FLAG antibody revealed a polypeptide band with an apparent mass of 111.6 kDa consistent with the _FLAG_ArnT_PhoA_ predicted mass (Fig. 4B). The CC118 strain expressing the fusion protein produced 400 U of alkaline phosphatase activity (Fig. 4C), which was comparable to that of the positive control (CC118 expressing Wzx_K367-PhoA_)^30^. Together, these experiments strongly support the conclusion that the C-terminal region of *B. cenocepacia* ArnT is oriented towards the periplasm.

**Figure 5:**
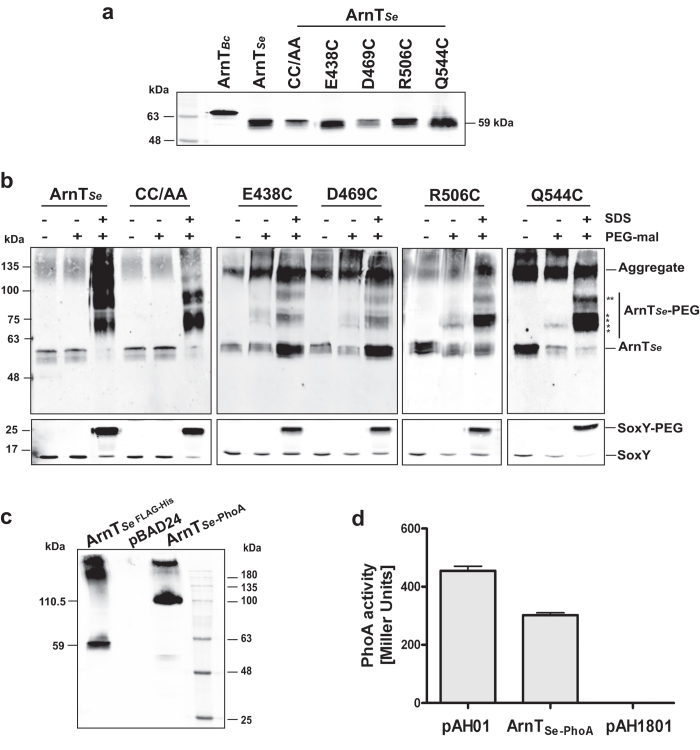
C-terminus of ArnT from S. enterica is located in the periplasm. (**a**) Immunoblot of total membranes isolated from *E. coli* DH5α containing plasmids expressing ArnT_Se_ cysteine replacements. The proteins were separated by SDS-PAGE and immunoblotting was performed using anti-FLAG. ArnT_Se-FLAG-10xHis_ was used as control. C148A-C149A (CC/AA) was used as a template to generate the cysteine-substitution in the C-terminal of ArnT_Se_. (**b**) PEGylated samples of ArnT_*Se*_ were prepared and analyzed as described in the legend to Fig. 2B. The positions of the molecular mass marker are indicated to the left. Asterisks indicate PEGylated ArnT_Se_ forms. ArnT_Se_ aggregates at the top of the western blots are attributed to the mild denaturing conditions. (**c**) Immunoblot of total membranes from *E. coli* CC118 cells expressing _FLAG_-ArnT_Se-PhoA_ performed with anti-FLAG. (**d**) Quantification of PhoA activity in CC118 *E. coli* expressing _FLAG-_ArnT_Se-PhoA_. Cells were processed as described in the legend to Fig. 4C using the same positive and negative controls. The values represent the mean ± SE from three independent experiments.

In a previous report^23^, the C-terminal region of the *S. enterica* ArnT was assigned to the cytosol without experimental confirmation and despite that a periplasmic location was predicted *in silico*. ArnT_Se_ has eight native cysteine residues, two of which (C148 and C149) were predicted to be in the second periplasmic loop and the remaining six cysteines embedded in transmembrane segments^23^. Therefore, we constructed a double replacement mutant, ArnT_C148A-C149A_, which was used to further introduce novel cysteines in the C-terminal domain without making a complete Cys-less version of ArnT_Se_. Novel cysteines replaced glutamic acid-438, aspartic acid-469, arginine-506, and glutamine-544 residues. Mutant proteins and parental ArnT_Se_ were detectable by immunoblotting (Fig. 5A). PEGylation was performed in whole cells with and without detergent. ArnT_E438C_, ArnT_D469C_, ArnT_R506C_ and ArnT_Q544C_ were PEGylated in non-denaturing conditions (Fig. 5B). Since all these residues were accessible to PEG-mal we concluded that they are exposed to the periplasmic face and not to the cytosolic space as previously proposed^23^. As a control, the cytoplasmic HA-SoxY protein was only PEGylated under SDS treatment (Fig. 5B), ruling out loss of cell membrane integrity during whole cell PEGylation. SDS treatment also showed PEGylation of the ArnT_Se_ derivatives under denaturing conditions (Fig. 5B, asterisk), as expected given that there were six remaining native cysteines in the protein. Importantly, the parental ArnT_Se_ was not PEGylated in absence of SDS (Fig. 5B), indicating that cysteines 148 and 149 are not exposed to the second periplasmic loop, as it was reported^23^.

Further, we investigated the location of the ArnT_Se_ C-terminus by constructing a PhoA fusion (_FLAG_ArnT_Se-PhoA_). Immunoblot of total membrane preparations reacted with anti-FLAG (Fig. 5C) identified a 110.5-kDa-polypeptide band as expected, indicating that ArnT_Se_ is correctly fused to the PhoA protein and that is also in the membrane fraction. Lysates of strain CC118 expressing the fusion protein displayed 350 U of alkaline phosphatase activity (Fig. 5D), which was comparable to the activity of the positive control cell lysates from CC118 expressing Wzx_K367-PhoA_^30^. Therefore, the ArnT_Se_ C-terminal domain is exposed towards the periplasm, as in ArnT_Bc_.

### Identification of functional amino acids in ArnT

We examined the functionality of ArnT_Bc_ cysteine replacement mutants by determining their ability to restore PmB resistance when expressed in the *B. cenocepacia* ∆*arnT lptG* suppressor mutant (Supplementary Table S1 online). Only proteins with replacements at tyrosine-43, lysine-69, arginine-254, and glutamic acid-493 residues, all of which are exposed to the periplasmic space (Fig. 1), failed to complement PmB resistance. Multiple sequence alignment revealed that tyrosine-43, lysine-69 (first periplasmic loop), arginine-254 (second periplasmic loop), and glutamic acid-493 (C-terminal segment) residues are highly conserved in ArnT proteins from different bacteria. Also, tyrosine-43 and lysine-69 residues are part of two distinct highly conserved motifs: ^42^RYA^44^ and ^66^YFEKP^70^ (Fig. 1 and Supplementary Figure S3A online), further suggesting that the identified residues could play a functional role.

Because loss of function in ArnT_Y43C_, ArnT_K69C_, ArnT_R254C_, and ArnT_E493C_ could also reflect structural modifications imposed by the non-native cysteine residue, we constructed alanine replacements at the same positions. The alanine replacement mutant proteins expressed at wild type levels and they also appeared in the membrane fraction (Supplementary Figure S3B online). To investigate whether the alanine replacement alters the local conformation of the loop, each residue before alanine-43, -69, -254, and -493 in ArnT_Y43A_, ArnT_K69A_, ArnT_R254A_, and ArnT_E493A_ proteins was replaced by cysteine. PEGylation assays using the resulting proteins with cysteine/alanine replacements (ArnT_R42C-Y43A_, ArnT_E68C-K69A_, ArnT_R253C-R254A_, and ArnT_D492C-E493A_) showed labeling of the neighboring cysteines under non-denaturing conditions (Supplementary Figure S3C online). Since the residues next to the alanine replacements are also accessible to PEG-mal, we conclude that it would unlikely that the alanine replacements cause dramatic changes in the local conformation of the protein at these sites.

To investigate the contribution of the mutated residues to ArnT activity we evaluated the PmB sensitivity of ∆*arnT* expressing the alanine replacement mutant proteins. The wild type strain producing LPS with l-Ara4N was extremely resistant to PmB with a minimal inhibitory concentration (MIC) greater than 1,024 μg ml^−1^, while the ∆*arnT* MIC was 0.064 μg ml^−1^ (16,000-fold difference)^13^. MICs for ∆*arnT* expressing ArnT_K69R_, ArnT_R254A_ and ArnT_E493A_ ranged from 8,000-fold reduction relative to wild type, while ∆*arnT* expressing ArnT_K69A_ and ArnT_Y43A_ had the same MIC as ∆*arnT* (Table 1), indicating that these replacements affected ArnT activity. Because alanine is a small amino acid typically used in replacements to prevent structural changes in proteins, we surmise that tyrosine-43, lysine-69, arginine-254 and glutamic acid-493 are involved in ArnT function.

Lysine-69, arginine-254, and glutamic acid-493 are charged amino acids. To investigate whether the charge or the nature of the amino acid is important for ArnT function we replaced lysine-69 with arginine and glutamic acid, arginine-254 with lysine and glutamic acid, and glutamic acid-493 with aspartic acid and lysine. All mutant proteins were detectable in whole cells by immunoblotting (Supplementary Figure S3D online). Neither lysine-69 nor arginine-254 replaced with opposite or same-charge amino acids resulted in proteins that could restore PmB resistance at MIC values similar to the wild type strain (Table 1). In contrast, ArnT containing glutamic acid-493 replaced by aspartic acid restored PmB resistance in ∆*arnT* at 128 μg ml^−1^ (Table 1), while the opposite charge substitution, ArnT_E493K_, led to a MIC of 0.064 μg ml^−1^, identical to that of ∆*arnT*. Together, these data suggest that tyrosine-43 and lysine-69 are needed for ArnT function, and also that positive and negative charges at position 254 and 493, respectively, are required for ArnT activity.

**Figure 6:**
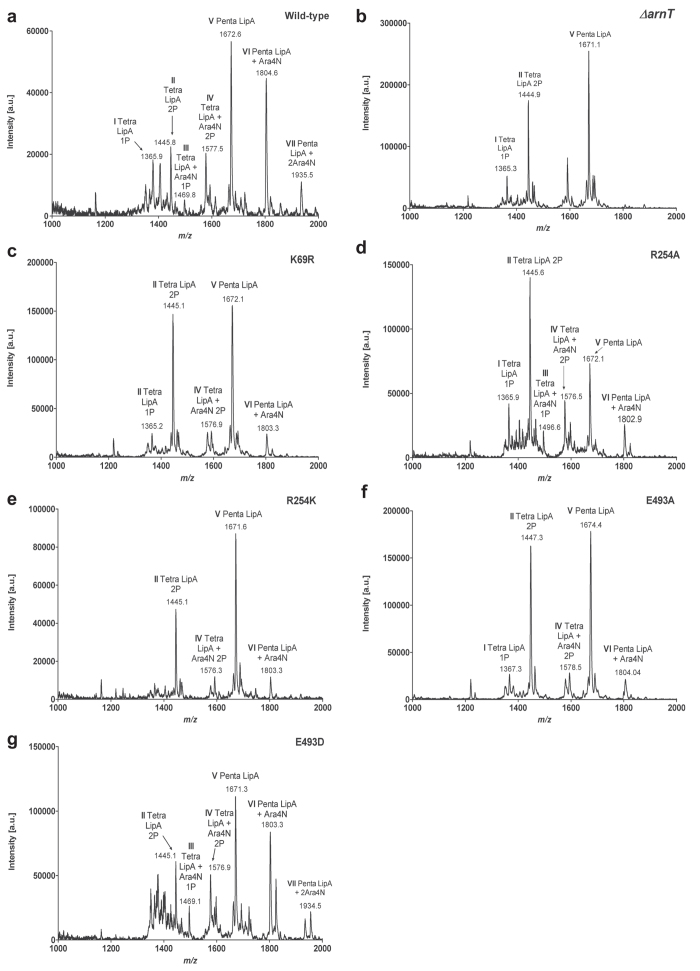
MALDI-TOF spectra of purified lipid A produced by arnT mutants. ∆*arnT* denotes strain MH55 (∆*arnT-arnBC*^+^ *lptG*^S^), which carries a *lptG* suppressor mutation (Table S1). Plasmids encoding ArnT proteins with various replacements are introduced into MH55 by triparental mating. The profiles represented were obtained using the negative ion mode. I-II Tetra-acylated lipid A with one or two phosphates molecules; III-IV, Tetra-acylated lipid A with one or two l-Ara4N molecules. V, Penta-acylated lipid A; VI-VII, Penta-acylated lipid A with one or two l-Ara4N molecules.

The effect of the amino acid replacement mutants on ArnT function was also evaluated by examining the lipid A structure using matrix-assisted laser desorption ionization-time of flight (MALDI-TOF) mass spectrometry in the negative ion mode. The mass spectrum from the wild-type sample showed the presence of l-Ara4N as indicated by the ion peaks corresponding to tetra-acylated lipid A with one l-Ara4N in the monophosphate (at *m/z* 1496.8) and diphosphate (at *m/z* 1577.5) forms and penta-acylated lipid with one and two l-Ara4N (at *m/z* 1804.6 and *m/z* 1935.5, respectively) (Fig. 6A), agreeing with the reported *B. cenocepacia* lipid A structure^13^,^31^. As expected, these peaks were absent in the ∆*arnT* lipid A spectrum (Fig. 6B and Table 1). Mass spectrometry analysis of lipid A from ∆*arnT* expressing ArnT_Y43A_, ArnT_K69A_, ArnT_K69E_, ArnT_R254E_ and ArnT_E493K_ did not reveal any peaks corresponding to glycoforms containing l-Ara4N (Table 1 and Supplementary Figure S4 online). Interestingly, l-Ara4N was present in ∆*arnT-arnBC*^*+*^containing, ArnT_K69R_, ArnT_R254A_, ArnT_R254K_, ArnT_E493A_, and ArnT_E493D_ (Fig. 6C-G and Table 1). Because MALDI-TOF does not provide quantitative assessments, we interpreted the presence of l-Ara4N as a result of reduced but not abolished ArnT enzyme activity. This interpretation agrees with the hypersensitivity to PmB of the strain expressing these mutant proteins (Table 1), suggesting insufficient amount of lipid A modification to promote wild type levels of resistance to PmB. We therefore conclude that tyrosine-43, lysine-69, arginine-254 and glutamic acid-493 residues are important for ArnT function.

To better support these conclusions, we employed the conditional *B. cenocepacia* mutant P_*rha*_::*arnT*, which only grows with rhamnose (permissive condition)^13^. Recombinant plasmids containing ArnT_Bc_ derivatives were introduced into *P*_*rha*_::a*rnT* and the resulting transformants examined for viability without rhamnose in the medium. Recombinant plasmids expressing ArnT_R254A_, ArnT_R254K_ and ArnT_E493D_ could rescue *P*_*rha*_::a*rnT* growth without rhamnose (Supplementary Figure S5 online). In contrast, vector control and plasmids containing ArnT_K69E_, ArnT_R254E_, ArnT_E493K_ and ArnT_E493A_, failed to rescue growth under the same condition. Bacteria containing ArnT_K69A_ and ArnT_K69R_ had moderate growth. These results agree with the lipid A structural analysis. Strains bearing ArnT_R254A_, ArnT_R254K_ and ArnT_E493D_ had l-Ara4N in the LPS, and they could rescue viability in absence of rhamnose. Nevertheless, the MIC value from these strains is very similar to ∆*arnT-arnBC*^*+*^ except for the strains with ArnT_E493D_, which behaves as the wild-type strain.

Together, these results indicate that ArnT with substitutions preserving the net charge of each residue were slightly functional, while substitutions reversing the charge caused loss of function. Therefore, the net charges in lysine-69 and arginine-254 are important for ArnT activity.

### Tyrosine-36, lysine-62 and glutamic acid-478 are also important for *S. enterica* ArnT

**Figure 7:**
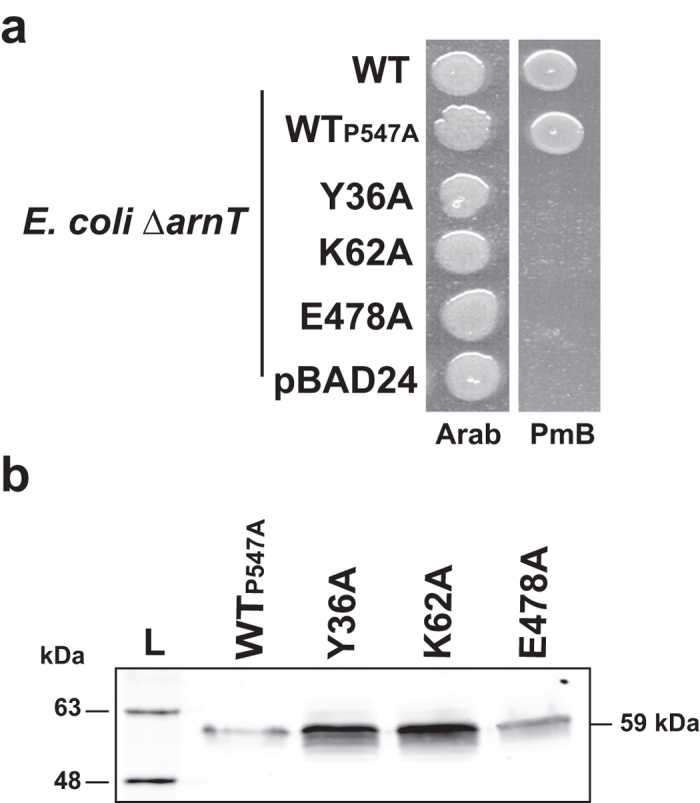
S. enterica ArnT shares the same functional residues than B. cenocepacia ArnT. (**a**) Complementation of *E. coli* ∆*arnT* with functional residues replaced with alanine from ArnT_Se_. WT, ArnT_P547A-FLAG-10xHis_ as a positive control; pBAD24 as a negative control. Strains were spotted in LB plate with 0.2% arabinose (Arab) or 0.2% arabinose plus PmB 2.5 μg/ml (PmB). (**b**) Total protein from cells expressing ArnT_Se_ derived proteins were separated by SDS-PAGE and detected with antibody anti-FLAG. L, molecular masses of protein standards.

An earlier report identified arginine-246 (corresponding arginine-254 of ArnT_Bc_) as being functionally important for the *S. enterica* ArnT^23^. We investigated whether ArnT_Se_ tyrosine-36, lysine-62, and glutamic acid-478 (corresponding to tyrosine-43, lysine-69, and glutamic acid-493 in ArnT_Bc_, respectively) are also required for ArnT_Se_ function. However, the ArnT_Se-FLAG-10xHis_ fusion resulted in a protein that could not be detected by immunoblot. This could be due to structural constrains associated with a proline at the C-terminal end of the protein, which were resolved by replacing proline-547 by alanine without altering ArnT function^32^. Therefore, we constructed a P547A ArnT_Se-FLAG-10xHis_ protein and the corresponding alanine replacements in tyrosine-36, lysine-62, and glutamic acid-478. The recombinant proteins were tested for their ability to restore PmB resistance in an *E. coli* Δ*arnT* mutant. The complementation experiment indicated that these proteins could not confer PmB resistance, suggesting that the mutated residues are also important for ArnT activity in *S. enterica* (Fig. 7A); the stability of these proteins was not affected as shown by immunoblotting (Fig. 7B). Therefore, tyrosine-36, lysine-62 and glutamic acid-478 play a crucial role in the function of the ArnT_Se_, demonstrating that the conserved amino acids in ArnT_Bc_ are also important in other ArnT family proteins.

## Discussion

Glycosylation of the lipid A phosphate groups with l-Ara4N, catalyzed by ArnT, is a strategy commonly used by bacteria to overcome the action of antimicrobial peptides such as PmB^33^. However, despite the growing number of ArnT homologs found in different bacteria to date very little is known on the mechanism of ArnT modification. We report in this work the membrane topology of *B. cenocepacia* and *S. enterica* ArnT proteins. Application of a topology prediction algorithm in combination with substituted cysteine scanning using PEGylation yielded an experimentally validated model for ArnT_Bc_ that suggests 13-transmembrane helices, two large periplasmic hydrophilic loops, and an extended C-terminus also located in the periplasm. Comparison of the *B. cenocepacia* ArnT model with an earlier model of ArnT from *S. enterica* reveals striking differences. Unlike ArnT_Bc_, the proposed ArnT_Se_ model suggested four periplasmic loops and placed the C-terminal end towards the cytosol^23^. Our PEGylation results do not support the earlier ArnT_Se_ model since two adjacent cysteines at positions 148 and 149, predicted to face the periplasm, were inaccessible to PEG-mal indicating they are buried in the membrane. In the previous model^23^, the ArnT_Se_ C-terminus was also assigned a cytosolic location without any experimental validation and despite bioinformatic data suggesting the opposite. In contrast, our PEGylation data and a positive alkaline phosphatase ArnT-PhoA C-terminal fusion strongly support that the C-terminal segment of ArnT_Se_ is in the periplasm. We therefore conclude that *B. cenocepacia* and *S. enterica* ArnT proteins share similar topology, as expected for proteins of the same family performing the same function.

We also identified putative functional residues in ArnT_Bc_ that are highly conserved in ArnT proteins from different bacteria. These include tyrosine-43, lysine-69, and arginine-254. Furthermore, a negative charged amino acid is required at position 493 (glutamic acid in ArnT_Bc_). All these putative functional residues reside on the periplasmic side of ArnT, as expected for an enzyme using substrates only available in the periplasm^15^,^17^. We propose that these residues may participate either in catalysis or binding of the undecaprenyl-l-Ara4N donor substrate. Tyrosine-43 and lysine-69 residues are within two highly conserved motifs in all ArnT family members: ^42^RYA^44^ and ^66^YFEKP^70^. The conservation of these motifs within the first periplasmic loop of ArnT_Bc_ suggest not only that they are functionally relevant but more importantly, that the members of ArnT family share similar features. Further, replacing tyrosine-43 of the RYA motif by alanine or inverting the polarity of the lysine-69 resulted in a nonfunctional protein. It is possible that the first periplasmic loop containing two highly conserved motifs could define a putative catalytic reaction center conserved in all ArnT proteins. Mutagenesis of the arginine-254 residue residing on the second periplasmic loop resulted in a protein with poor enzymatic activity. Agreeing with this conclusion, an arginine from ArnT_Se_ corresponding to ArnT_Bc_ arginine-254 was also reported to be important for the activity of this enzyme^23^. Our data also suggest that the ArnT_Se_ second periplasmic loop, between amino acids 234 and 256, is required for function, as well as a C-terminal glutamic acid-493. Replacing this residue by aspartic acid, to maintain the same charge, resulted in a protein retaining the ability to incorporate l-Ara4N to lipid A and supports the notion that the ArnT C-terminus is also involved in the overall activity of this enzyme. However, our assignment of the functional residues has to remain tentative until validation by further biochemical investigations using purified protein, substrates, and acceptor molecules.

The mechanism of transfer of l-Ara4N to LPS remains to be elucidated. Superficially, ArnT proteins are analogous to O-antigen ligases and oligosaccharyl transferases. These proteins perform the enzymatic reaction on the periplasmic side of the membrane, and involve a metal ion-independent inverting glycosyltransfer reaction using undecaprenyl-bisphosphate-linked saccharide precursors^34^. In contrast, bacteriophage-encoded glucosyltransferase proteins, which are responsible for the periplasmic transfer of glucose to the LPS O antigen moiety^35^,^36^, utilize a lipid phosphate-activated glucosyl residue (Und-P-glucose)^37^. ArnT proteins also utilize undecaprenyl-phosphate-linked saccharides, and the reaction occurs at the periplasmic site of the membrane^17^. Further, there is an inversion of the stereochemistry in the reaction since the substrate is Und-P-α-l-Ara4N^17^, but l-Ara4N is β-linked to the phosphate groups of the lipid A at 4’ and 1 positions^31^, suggesting ArnT proteins are also inverting glycosyltransferases. Given these similarities with other glycan ligases, it is reasonable to find that key functional residues are located on the periplasmic exposed side of ArnT.

In this work, we demonstrate that ArnT proteins from *B. cenocepacia* and *S. enterica* share topology and functional residues. In *B. cenocepacia,* however, l-Ara4N is added to lipid A and the d*-*glycero-d-*talo*-octo*-*2*-*ulosonic acid inner core sugar, modifications that confer extremely high resistance to PmB^13^. In contrast, *S. enterica* and *E. coli* ArnT transfer l-Ara4N only to lipid A and comparatively these strains present much lower levels of PmB resistance^31^. The topological similarities and functionally conserved residues between ArnT_Bc_ and ArnT_Se_ suggest that both proteins have more in common that one would expect if they had different specificities. However, we cannot exclude the possibility that another region in ArnT_Bc_ is responsible for specific recognition of the *Burkholderia* LPS, making this protein different than that of *E. coli*, *Salmonella*, and other bacteria. Alternatively, a distinct l-Ara4N transferase could be required in *B. cenocepacia* to add l-Ara4N to the inner core d*-*glycero-d-*talo-*octo*-*2*-*ulosonic acid after ArnT transfers l-Ara4N to the lipid A. Further research is needed to investigate these possibilities and examine the sequential process of l-Ara4N incorporation to lipid A and inner core in detail.

In summary, this study provides experimental evidence that ArnT proteins from *B. cenocepacia* and *S. enterica*, which play key roles in remodeling LPS, share topological and functional similarities that also extend to other members of the ArnT family and provide insights for further investigation of ArnT proteins and the elucidation of their catalytic mechanism.

## Methods

### Bacterial strains and growth conditions

Bacterial strains and plasmids are listed in Supplementary Table S1 online. Growth medium was supplemented as needed with the following chemicals (final concentrations): 100 μg ampicillin ml^−1^, 30 μg chloramphenicol ml^−1^, 50 μg trimethoprim ml^−1^, 25 μg gentamicin ml^−1^, 10 μg PmB ml^−1^, 0.2% l-arabinose (w/v), and 0.2% l-rhamnose (w/v). Bacteria were grown aerobically at 37 °C in Luria-Bertani (LB) medium (Difco). For recombinant protein expression, bacteria were grown overnight in 5 mL of LB, diluted to *A*_600_ of 0.1, and incubated at 37 °C for 2 h until cultures reached *A*_600_ of 0.5, at which point l-arabinose was added and cells incubated for 3 h at 37 °C.

### Construction of plasmids expressing parental and mutant ArnT proteins

A plasmid encoding ArnT_FLAG-10xHis_ was constructed by PCR amplification of a 1.7-kb fragment using *B. cenocepacia* K56-2 genomic DNA and primers 6269/6270 (Supplementary Table S2 online). The fragment was digested with *Eco*RI and *Sal*I and ligated into the same sites of pXR1^34^. This plasmid is a modified pBAD24 containing an oligonucleotide sequence encoding a FLAG plus 10x-His tag, which was placed adjacent to the multiple cloning site for construction of in-frame C-terminally FLAG-10xHis-tagged proteins. The resulting plasmid, designated pFT1, expressed ArnT_FLAG-10xHis_ under the control of the arabinose-inducible promoter. pFT1 was used as template to replace the native cysteines of ArnT (residues 154 and 176) with alanine by sequential site-direct mutagenesis using primers 6238/6239, and 6240/6241 (Supplementary Table S2 online). The plasmid encoding the cysteineless ArnT_FLAG-10xHis_ was named pFT7. For sulfhydryl labeling experiments, novel cysteines were introduced in pFT7 by PCR using primers containing the desired mutations and *Pfu* AD polymerase (Stratagene). *Dpn*I was added to PCR reactions for overnight digestion of parental plasmid DNA at 37 °C. The resulting DNA was introduced into *E. coli* DH5α by CaCl_2_ transformation. Resulting plasmids were confirmed by sequencing with primers 252, 258 and 6385 (Supplementary Table S2 online).

### Alkaline phosphatase (PhoA) fusions and alkaline phosphatase assay

pFT4 and pFT15, expressing _FLAG_ArnT_Bc-PhoA_ and _FLAG_ArnT_Se-PhoA_, respectively, were constructed by PCR amplification of a 1.7-kb fragment from pFT1 and pMH494 with 6421/6716 and 7178/7182 primer pairs, respectively (Supplementary Table S2 online). The amplicons were digested with *Xba*I and *Sal*I and ligated into these sites in pAH18^30^. The ligation mixture was introduced by transformation into *E. coli* CC118 cells, and transformants plated on LB-ampicillin containing 5-bromo-4-chloro-3-indolylphosphate (60 μg ml^−1^). Transformants were screened for blue colony phenotype denoting PhoA activity. To quantify alkaline phosphatase activity, overnight cultures were diluted 1:100 and grown for 4 h at 37 °C in LB ampicillin/0.2% arabinose, harvested, lysed, and assayed as described^30^.

### Cloning and expression of *arnT* from *S. enterica*

The *S. enterica arnT* gene was isolated by amplification of a 1.6-kb fragment using pMH494 as template and primers 6475/6476. The fragment was digested with *Eco*RI and *Sal*I and ligated into the same sites in pFT1 to replace *arnT* from *B. cenocepacia* by *S. enterica arnT*. This construct, named pFT54, expressed ArnT_Se_ with a C-terminal FLAG-10xHis tag, and was used to introduce novel cysteines by site-directed mutagenesis. For complementation of *E. coli* ∆*arnT*, alanine replacements were introduced in pFT143 (encoding *S. enterica* ArnT_P547A_).

### Topological model for ArnT

Two prediction methods were used to analyze the *in silico* topology of ArnT: PolyPhobious^38^ and TMHMM 2.0^39^. The ArnT models were displayed using TMRPred2D^40^. Multiple-sequence alignments of ArnT homologues were performed with ClustalW ( http://www.ebi.ac.uk/clustalw/).

### Complementation of ArnT function in *B. cenocepacia*

Plasmids expressing the ArnT replacement mutant proteins were subcloned into the *Nde*I-*Hin*dIII site of pSCrhaB2, and the recombinant constructs introduced in the ∆*arnT-arnBC*^*+*^suppressor strain by triparental mating^41^,^42^. The ability of each construct to restore PmB resistance indicated ArnT function. Strains were grown overnight with 0.4% (w/v) rhamnose (to induce protein expression), diluted to 10^−1^, 10^−2^, 10^−3^, 10^−4^ and 10^−5^ and 4 μl of each dilution plated onto LB plates supplemented with 0.4% rhamnose and 10 μg ml^−1^ PmB.

### Membrane preparations and immunoblotting

Bacterial pellets were resuspended in 50 mM Tris-HCl, pH 8.0, with protease inhibitors and lysed at 10, 000 PSI using a cell disruptor (Constant Systems, Kennesaw, GA). Cell debris was pelleted by centrifugation at 15,000 × *g* for 15 min at 4 °C, and the clear supernatant was centrifuged at 40,000 × *g* for 30 min at 4 °C. The pellet, containing total membranes, was suspended 50 mM Tris-HCl, pH 8.0. Protein concentration was determined by the Bradford assay (Bio-Rad). Proteins were separated by SDS-PAGE on a 12% or 18% gel (if the experiment included detection of HA-SoxY) and transferred to a nitrocellulose membrane. Immunoblots were probed with anti-FLAG and anti-HA (Sigma) mouse monoclonal antibodies. Secondary anti- mouse Alexa fluor-680 IgG antibody (Invitrogen) was used to detect fluorescence using an Odyssey infrared imaging system (LI-COR Biosciences).

### Sulfhydryl labeling

Cells were cultured in 20 ml of LB broth at 37 °C from an initial OD_600_ of 0.2 to an OD_600_ of 0.5 at which time arabinose was added to a final concentration of 0.2% (w/v) for induction of ArnT_FLAG-10xHis_ protein expression, and growth continued for 3 h. Bacterial cells were harvested by centrifugation at 16,000 × *g* and washed with HEPES/MgCl_2_ buffer (50 mM HEPES, pH 6.8; 5 mM MgCl_2_). Cell pellets were resuspended in 0.3 ml of HEPES/MgCl_2_ buffer and divided in three portion: 0.1 ml of cell suspension was incubated with 1 mM methoxypolyethylene glycol maleimide (PEG-Mal) at room temperature for 1 h with 50 mM EDTA for *B. cenocepacia* ArnT and 5 mM EDTA for *S. enterica* ArnT; 0.1 ml was treated with 2% SDS prior to labeling, and 0.1 ml was incubated with buffer as control. The sulfhydryl labeling reaction was stopped with 45 mM of dithiothreitol for 10 min and mixed with 3X dye buffer (50 mM Tris-HCl [pH 6.8], 2% SDS, 10% glycerol, and 0.1% bromophenol blue), and incubated for 30 min at 45 °C. Mild denaturation conditions were used to optimize ArnT detection, as these conditions work best for membrane proteins^43^. The crude membrane fraction was resuspended in 50 μl of HEPES/ MgCl_2_ buffer supplemented with protease inhibitors. 25 μl were labeled with 1 mM PEG-Mal at room temperature for 1 h. The reaction was quenched by adding 45 mM of dithiothreitol for 10 min, and centrifuged at 16,000 × g for 1 min. Pellets were suspended in 50 μl of HEPES/MgCl_2_ buffer with protease inhibitor. Control samples were incubated with buffer alone. Protein was extracted and processed as described above.

### Thiol-specific chemical blocking with N-ethylmaleimide (NEM)

Cell suspensions in HEPES/MgCl_2_ buffer (0.1 ml) were incubated with 5mM N-ethylmaleimide (NEM) for 30 min at room temperature, and centrifuged at 16,000 × g for 1 min. Pellets were washed twice with 1 ml of iced HEPES/MgCl_2_ buffer. The samples were suspended in 200 μl of HEPES/MgCl_2_ buffer, and incubated with 2% SDS and 1 mM PEG-Mal for 1 h at room temperature. Samples were quenched with 45 mM of dithiothreitol for 10 min. Protein extraction was performed as described above.

### MIC determination

The MIC was determined on solid media using PmB E-test strips. Overnight cultures, were diluted to an *A*_600_ of 0.004 in sterile PBS and used to streak LB agar plates using a sterile cotton swab. E-test strips containing PmB were added and plates where incubated for 24 h at 37 °C. MIC was defined as the lowest concentration of PmB to show growth inhibition.

### Lipid A isolation and mass spectrometry

L-Ara4N modifications of lipid A were identified by mass spectrometry (MS) using matrix-assisted laser desorption/ionization-time of flight (MALDI-TOF). Cells were grown overnight in LB medium (100 ml) supplemented with 50-μg ml^−1^ trimethoprim and 0.4% rhamnose. Cultures were centrifuged (10,000 × *g*), washed twice with phosphate buffer (10 mM Na_2_HPO_4_, 1.7 mM KH_2_PO_4_) and freeze-dried. Lipid A was extracted from lyophilized cells and desalted, as previously described^44^. For MS analysis, dihydroxybenzoic acid (DHB) (Sigma Chemical Col, St. Louis, MO) was used as a matrix and prepared to saturation in acetonitrile: 0.1% triflouroacetic acid (1:2 v/v). Two-microliters of sample was loaded on the target, dried, and covered by 1 μl of DHB. The target was inserted in a Bruker Autoflex MALDI-TOF spectrometer. Data acquisition and analysis were performed using the Flex Analysis software.

## Additional Information

**How to cite this article**: Tavares-Carreón, F. *et al.* *Burkholderia cenocepacia and Salmonella enterica* ArnT proteins that transfer 4-amino-4-deoxy-L-arabinose to lipopolysaccharide share membrane topology and functional amino acids. *Sci. Rep.* **5**, 10773; doi: 10.1038/srep10773 (2015).

## Supplementary Material

Supplementary Information

## Figures and Tables

**Table 1:** PmB MIC values of cells expressing ArnT mutant proteins unable to complement the ∆*arnT-arnBC* ^+^ *lptG*_D31H_ (∆*arnT*) suppressor mutant.

**Residue**	**MIC (μg ml**^−**1**^)	**Fold difference**	**Presence of l-Ara4N in lipid A**
ArnT	>1,024	1	Yes
∆*arnT-arnBC*^*+*^	0.064	16,000	No
ArnT_Y43A_	0.064	16,000	No
ArnT_K69A_	0.064	16,000	No
ArnT_K69R_	0.125	8,192	Yes
ArnT_K69E_	0.094	10,894	No
ArnT_R254A_	0.125	8,192	Yes
ArnT_R254K_	0.064	16,000	Yes
ArnT_R254E_	0.064	16,000	No
ArnT_E493A_	0.125	8,192	Yes
ArnT_E493D_	128	8	Yes
ArnT_E493K_	0.064	16,000	No

MICs were determined by E-Test. The presence of l-Ara4N in the lipid A was determined by MS-MALDI-TOF using the negative ion mode, as described in Methods.

## References

[b1] Govan J. R. & Deretic V. Microbial pathogenesis in cystic fibrosis: mucoid *Pseudomonas aeruginosa* and *Burkholderia cepacia*. Microbiol Rev 60, 539–574 (1996). 8840786 10.1128/mr.60.3.539-574.1996 PMC239456

[b2] Fang K. *et al.* Exploring the metabolic network of the epidemic pathogen *Burkholderia cenocepacia* J2315 via genome-scale reconstruction. BMC Syst Biol 5, 83 (2011). 21609491 10.1186/1752-0509-5-83 PMC3123600

[b3] Leitão J. H. *et al.* Variation of the antimicrobial susceptibility profiles of *Burkholderia cepacia* complex clonal isolates obtained from chronically infected cystic fibrosis patients: a five-year survey in the major Portuguese treatment center. Eur J Clin Microbiol Infect Dis 27, 1101–1111 (2008). 18600352 10.1007/s10096-008-0552-0

[b4] Loutet S. A. & Valvano M. A. Extreme antimicrobial peptide and polymyxin B resistance in the genus *Burkholderia*. Front Cell Infect Microbiol 1, 6 (2011). 22919572 10.3389/fcimb.2011.00006 PMC3417367

[b5] Loutet S. A., Di Lorenzo F., Clarke C., Molinaro A. & Valvano M. A. Transcriptional responses of *Burkholderia cenocepacia* to polymyxin B in isogenic strains with diverse polymyxin B resistance phenotypes. BMC Genomics 12, 472 (2011). 21955326 10.1186/1471-2164-12-472 PMC3190405

[b6] Ortega X., Silipo A., Saldias M. S., Bates C. C., Molinaro A. & Valvano M. A. Biosynthesis and structure of the *Burkholderia cenocepacia* K56-2 lipopolysaccharide core oligosaccharide: truncation of the core oligosaccharide leads to increased binding and sensitivity to polymyxin B. J Biol Chem 284, 21738–21751 (2009). 19525227 10.1074/jbc.M109.008532 PMC2755896

[b7] Loutet S. A., Mussen L. E., Flannagan R. S. & Valvano M. A. A two-tier model of polymyxin B resistance in *Burkholderia cenocepacia*. Environ Microbiol Rep 3, 278–285 (2011). 23761261 10.1111/j.1758-2229.2010.00222.x

[b8] Loutet S. A., Flannagan R. S., Kooi C., Sokol P. A. & Valvano M. A. A complete lipopolysaccharide inner core oligosaccharide is required for resistance of *Burkholderia cenocepacia* to antimicrobial peptides and bacterial survival *in vivo*. J Bacteriol 188, 2073–2080 (2006). 16513737 10.1128/JB.188.6.2073-2080.2006 PMC1428139

[b9] Silipo A. *et al.* The complete structure and pro-inflammatory activity of the lipooligosaccharide of the highly epidemic and virulent gram-negative bacterium *Burkholderia cenocepacia* ET-12 (strain J2315). Chemistry 13, 3501–3511 (2007). 17219455 10.1002/chem.200601406

[b10] Ortega X., Silipo A., Saldías M. S., Bates C. C., Molinaro A. & Valvano M. A. Biosynthesis and structure of the *Burkholderia cenocepacia* K56-2 lipopolysaccharide core oligosaccharide: truncation of the core oligosaccharide leads to increased binding and sensitivity to polymyxin B. J Biol Chem 284, 21738–21751 (2009). 19525227 10.1074/jbc.M109.008532 PMC2755896

[b11] Vinion-Dubiel A. D. & Goldberg J. B. Lipopolysaccharide of *Burkholderia cepacia* complex. J Endotoxin Res 9, 201–213 (2003). 12935351 10.1179/096805103225001404

[b12] Ortega X. P. *et al.* A putative gene cluster for aminoarabinose biosynthesis is essential for *Burkholderia cenocepacia* viability. J Bacteriol 189, 3639–3644 (2007). 17337576 10.1128/JB.00153-07 PMC1855895

[b13] Hamad M. A., Di Lorenzo F., Molinaro A. & Valvano M. A. Aminoarabinose is essential for lipopolysaccharide export and intrinsic antimicrobial peptide resistance in *Burkholderia cenocepacia*. Mol Microbiol 85, 962–974 (2012). 22742453 10.1111/j.1365-2958.2012.08154.x

[b14] El-Halfawy O. M. & Valvano M. A. Chemical communication of antibiotic resistance by a highly resistant subpopulation of bacterial cells. PLoS One 8, e68874 (2013). 23844246 10.1371/journal.pone.0068874 PMC3700957

[b15] Raetz C. R., Reynolds C. M., Trent M. S. & Bishop R. E. Lipid A modification systems in gram-negative bacteria. Annu Rev Biochem 76, 295–329 (2007). 17362200 10.1146/annurev.biochem.76.010307.145803 PMC2569861

[b16] Yan A., Guan Z. & Raetz C. R. An undecaprenyl phosphate-aminoarabinose flippase required for polymyxin resistance in *Escherichia coli*. J Biol Chem 282, 36077–36089 (2007). 17928292 10.1074/jbc.M706172200 PMC2613183

[b17] Trent M. S., Ribeiro A. A., Lin S., Cotter R. J. & Raetz C. R. An inner membrane enzyme in *Salmonella* and *Escherichia coli* that transfers 4-amino-4-deoxy-L-arabinose to lipid A: induction on polymyxin-resistant mutants and role of a novel lipid-linked donor. J Biol Chem 276, 43122–43131 (2001). 11535604 10.1074/jbc.M106961200

[b18] Miller S. I., Ernst R. K. & Bader M. W. LPS, TLR4 and infectious disease diversity. Nat Rev Microbiol 3, 36–46 (2005). 15608698 10.1038/nrmicro1068

[b19] Raetz C. R. & Whitfield C. Lipopolysaccharide endotoxins. Annu Rev Biochem 71, 635–700 (2002). 12045108 10.1146/annurev.biochem.71.110601.135414 PMC2569852

[b20] Wang X., Ribeiro A. A., Guan Z. & Raetz C. R. Identification of undecaprenyl phosphate-beta-D-galactosamine in *Francisella novicida* and its function in lipid A modification. Biochemistry 48, 1162–1172 (2009). 19166327 10.1021/bi802211k PMC2701470

[b21] Marr N., Tirsoaga A., Blanot D., Fernandez R. & Caroff M. Glucosamine found as a substituent of both phosphate groups in *Bordetella* lipid A backbones: role of a BvgAS-activated ArnT ortholog. J Bacteriol 190, 4281–4290 (2008). 18424515 10.1128/JB.01875-07 PMC2446747

[b22] Rolin O. *et al.* Enzymatic modification of lipid A by ArnT protects *Bordetella bronchiseptica* against cationic peptides and is required for transmission. Infect Immun 82, 491–499 (2014). 24478065 10.1128/IAI.01260-12 PMC3911393

[b23] Impellitteri N. A., Merten J. A., Bretscher L. E. & Klug C. S. Identification of a functionally important loop in *Salmonella typhimurium* ArnT. Biochemistry 49, 29–35 (2010). 19947657 10.1021/bi901572f PMC2805049

[b24] Koch S., Fritsch M. J., Buchanan G. & Palmer T. *Escherichia coli* TatA and TatB proteins have N-out, C-in topology in intact cells. J Biol Chem 287, 14420–14431 (2012). 22399293 10.1074/jbc.M112.354555 PMC3340247

[b25] Nasie I., Steiner-Mordoch S. & Schuldiner S. Topology determination of untagged membrane proteins. Methods Mol Biol 1033, 121–130 (2013). 23996174 10.1007/978-1-62703-487-6_8

[b26] Bogdanov M., Zhang W., Xie J. & Dowhan W. Transmembrane protein topology mapping by the substituted cysteine accessibility method (SCAM(TM)): application to lipid-specific membrane protein topogenesis. Methods 36, 148–171 (2005). 15894490 10.1016/j.ymeth.2004.11.002 PMC4104023

[b27] Rivera-Ordaz A. *et al.* The sodium/proline transporter PutP of *Helicobacter pylori*. PLoS One 8, e83576 (2013). 24358297 10.1371/journal.pone.0083576 PMC3866251

[b28] Kuper C. & Jung K. CadC-mediated activation of the cadBA promoter in *Escherichia coli*. J Mol Microbiol Biotechnol 10, 26–39 (2005). 16491024 10.1159/000090346

[b29] Haardt M. & Bremer E. Use of phoA and lacZ fusions to study the membrane topology of ProW, a component of the osmoregulated ProU transport system of Escherichia coli. J Bacteriol 178, 5370–5381 (1996). 8808924 10.1128/jb.178.18.5370-5381.1996 PMC178353

[b30] Marolda C. L. *et al.* Membrane topology and identification of critical amino acid residues in the Wzx O-antigen translocase from *Escherichia coli* O157:H4. J Bacteriol 192, 6160–6171 (2010). 20870764 10.1128/JB.00141-10 PMC2981206

[b31] Silipo A. *et al.* Complete structural characterization of the lipid A fraction of a clinical strain of *B. cepacia* genomovar I lipopolysaccharide. Glycobiology 15, 561–570 (2005). 15610978 10.1093/glycob/cwi029

[b32] Bretscher L. E., Morrell M. T., Funk A. L. & Klug C. S. Purification and characterization of the L-Ara4N transferase protein ArnT from *Salmonella typhimurium*. Protein Expr Purif 46, 33–39 (2006). 16226890 10.1016/j.pep.2005.08.028

[b33] Needham B. D. & Trent M. S. Fortifying the barrier: the impact of lipid A remodelling on bacterial pathogenesis. Nat Rev Microbiol 11, 467–481 (2013). 23748343 10.1038/nrmicro3047 PMC6913092

[b34] Ruan X., Loyola D. E., Marolda C. L., Perez-Donoso J. M. & Valvano M. A. The WaaL O-antigen lipopolysaccharide ligase has features in common with metal ion-independent inverting glycosyltransferases. Glycobiology 22, 288–299 (2012). 21983211 10.1093/glycob/cwr150

[b35] Korres H. & Verma N. K. Topological analysis of glucosyltransferase GtrV of *Shigella flexneri* by a dual reporter system and identification of a unique reentrant loop. J Biol Chem 279, 22469–22476 (2004). 15028730 10.1074/jbc.M401316200

[b36] Korres H. & Verma N. K. Identification of essential loops and residues of glucosyltransferase V (GtrV) of *Shigella flexneri*. Mol Membr Biol 23, 407–419 (2006). 17060158 10.1080/09687860600849853

[b37] Nikaido K. & Nikaido H. Glucosylation of lipopolysaccharide in *Salmonella*: biosynthesis nof O antigen factor n12 2 . II. Structure of the lipid intermediate. J Biol Chem 246, 3912–3919 (1971). 4327192

[b38] Käll L., Krogh A. & Sonnhammer E. L. An HMM posterior decoder for sequence feature prediction that includes homology information. Bioinformatics 21 (Suppl 1), i251–257 (2005). 15961464 10.1093/bioinformatics/bti1014

[b39] Sonnhammer E. L., von Heijne G. & Krogh A. A hidden Markov model for predicting transmembrane helices in protein sequences. Proc Int Conf Intell Syst Mol Biol 6, 175–182 (1998). 9783223

[b40] Spyropoulos I. C., Liakopoulos T. D., Bagos P. G. & Hamodrakas S. J. TMRPres2D: high quality visual representation of transmembrane protein models. Bioinformatics 20, 3258–3260 (2004). 15201184 10.1093/bioinformatics/bth358

[b41] Craig F. F., Coote J. G., Parton R., Freer J. H. & Gilmour N. J. A plasmid which can be transferred between *Escherichia coli* and *Pasteurella haemolytica* by electroporation and conjugation. J Gen Microbiol 135, 2885–2890 (1989). 2693589 10.1099/00221287-135-11-2885

[b42] Figurski D. H. & Helinski D. R. Replication of an origin-containing derivative of plasmid RK2 dependent on a plasmid function provided *in trans*. Proc Natl Acad Sci U S A 76, 1648–1652 (1979). 377280 10.1073/pnas.76.4.1648 PMC383447

[b43] Ruan X. & Valvano M. A. *In vitro* O-antigen ligase assay. Methods Mol Biol 1022, 185–197 (2013). 23765663 10.1007/978-1-62703-465-4_15

[b44] El Hamidi A., Tirsoaga A., Novikov A., Hussein A. & Caroff M. Microextraction of bacterial lipid A: easy and rapid method for mass spectrometric characterization. J Lipid Res 46, 1773–1778 (2005). 15930524 10.1194/jlr.D500014-JLR200

