# Supplementary material for: Burkholderia cenocepacia and Salmonella enterica ArnT proteins that transfer 4-amino-4-deoxy-l-arabinose to lipopolysaccharide share membrane topology and functional amino acids
Source: Sci Rep. 2015 Jun 1;5:10773. doi: 10.1038/srep10773 (PMC5377068; doi:10.1038/srep10773)
Supplement: Supplementary Information [file srep10773-s1.pdf]

SUPPLEMENTARY INFORMATION

Burkholderia cenocepacia **and** ***Salmonella enterica*** **ArnT proteins that transfer 4-amino-4-*deoxy*-L-arabinose to lipopolysaccharide share membrane topology and functional amino acids**

Faviola Tavares-Carreon[1], Kinnari B. Patel[2] and Miguel A. Valvano[1,2*]

[1] Centre for Human Immunology, Department of Microbiology and Immunology, University of Western Ontario, London, Ontario, Canada, N6A 5C1; [2] Centre for Infection and Immunity, Queen's University Belfast, Belfast, United Kingdom, BT9 5AE

* Correspondence and requests for materials should be addressed to M.A.V (m.valvano@qub.ac.uk)

Supplementary figures

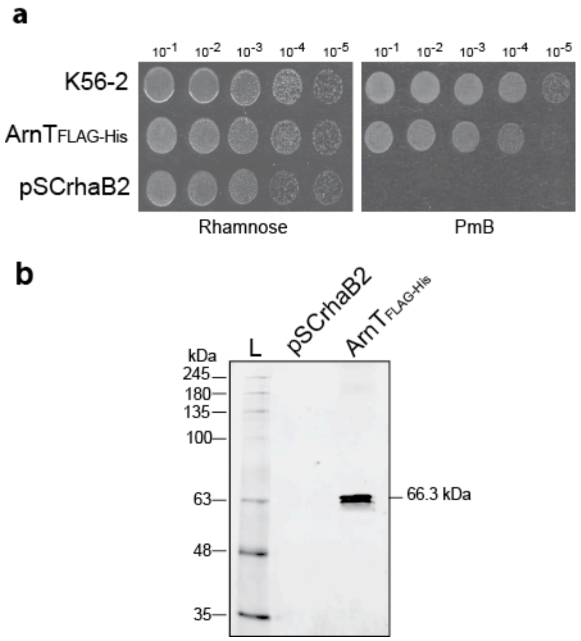

FIGURE S1 | Polymyxin B resistant phenotype is restored by ArnT$_{FLAG+10xHis}$ in Δ*arnT-arnBC*$^{+}$ carrying the lptG$_{D31H}$ suppressor mutation. (a) The strain Δ*arnT-arnBC*$^{+1}$ was transformed with pFT3 encoding ArnT$_{FLAG+10xHis}$ under the control of the rhamnose inducible promoter or pSCrhaB2 (vector control). K556-2 is the parental strains carrying the wild-type *arnT* (positive control). 10-fold serial dilutions of the transformants were spotted on LB supplemented with 0.4% of rhamnose and 10-µg ml^{-1} PmB. (b) Total membrane preparation from Δ*arnT-arnBC*$^{+}$ expressing ArnT$_{FLAG-10xHis}$ and pSCrhaB2 as a vector control. Ten µg of protein was analyzed by SDS-PAGE, and the immunoblot was probed with the antibody anti-FLAG. L, BLUeye prestained protein ladder

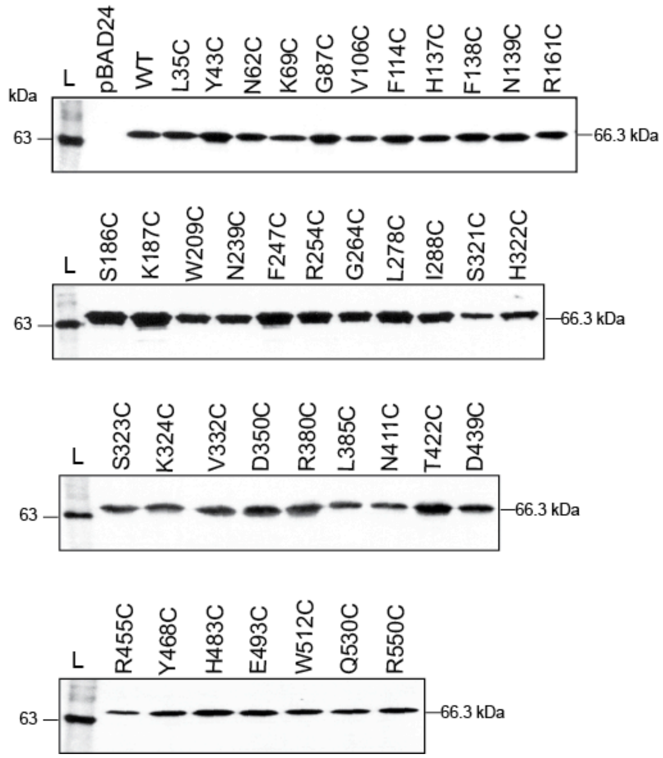

FIGURE S2 | Protein expression of ArnT cysteine replacement. $ArnT_{FLAG\text{-}10xHis}$ cysteine derivatives were expressed from the arabinose-inducible vector pBAD24. Twenty µl of total protein preparations from DH5α cells were separated by 12% SDS-PAGE and the ArnT mutant proteins detected by immunoblot with an anti-FLAG monoclonal antibody.

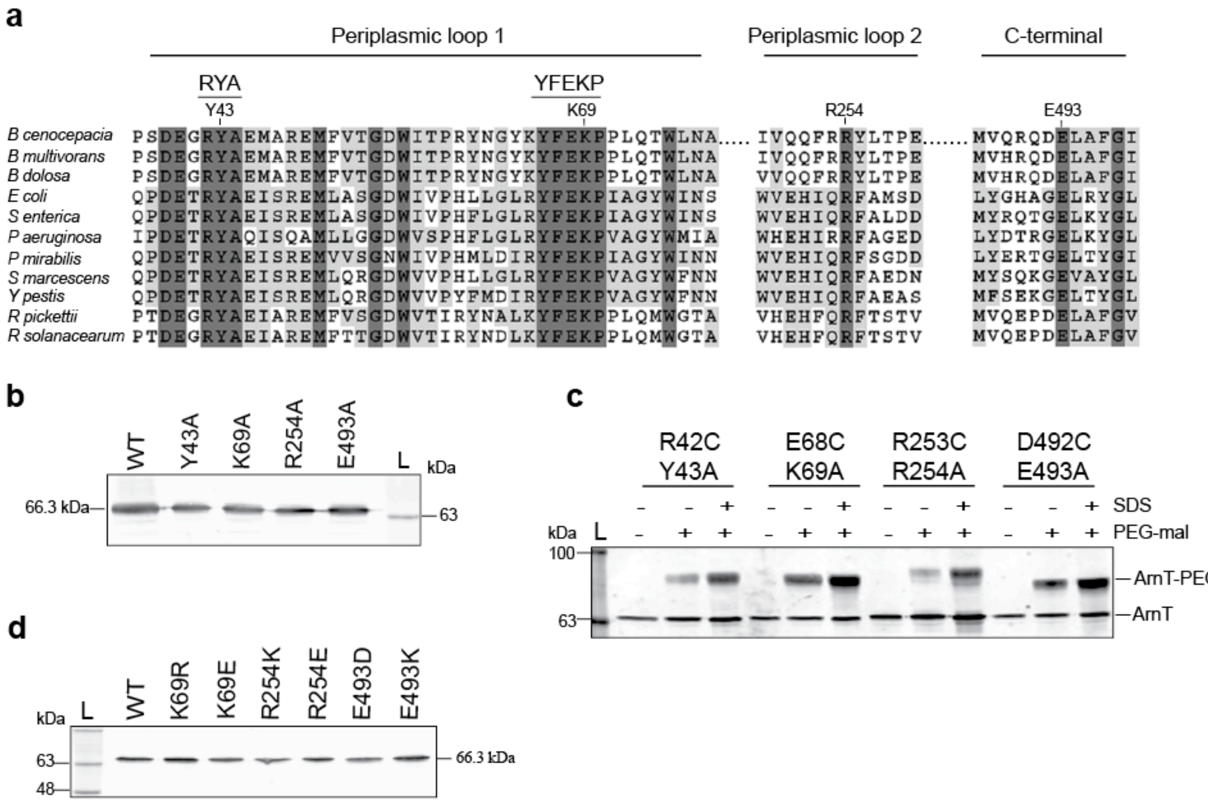

FIGURE S3 | Highly conserved residues of ArnT are required for activity. (a) alignment of partial sequences of ArnT from different bacteria showing RYA and YFEKP motifs and highly conserved Y43, K69, R254, and E493. (b-d) Immunoblot of ArnT proteins containing replacements in functional residues detected anti-FLAG. (c) *E. coli* DH5α carrying plasmids encoding ArnT cysteine replacements were grown to mid-exponential phase and protein expression induced with 0.2% L-arabinose. Cells were harvest and resuspend in 0.3 ml of HEPES/$MgCl_2$ buffer. 0.1 ml of cell suspension was incubated with buffer alone or 1 mM PEG-mal with or without 2% SDS for 1h at room temperature. Reactions were quenched with 45 mM DTT, and proteins were separated by SDS-PAGE and transferred to nitrocellulose membrane. ArnT derivatives were detected with anti-FLAG. L, molecular masses of protein standards.

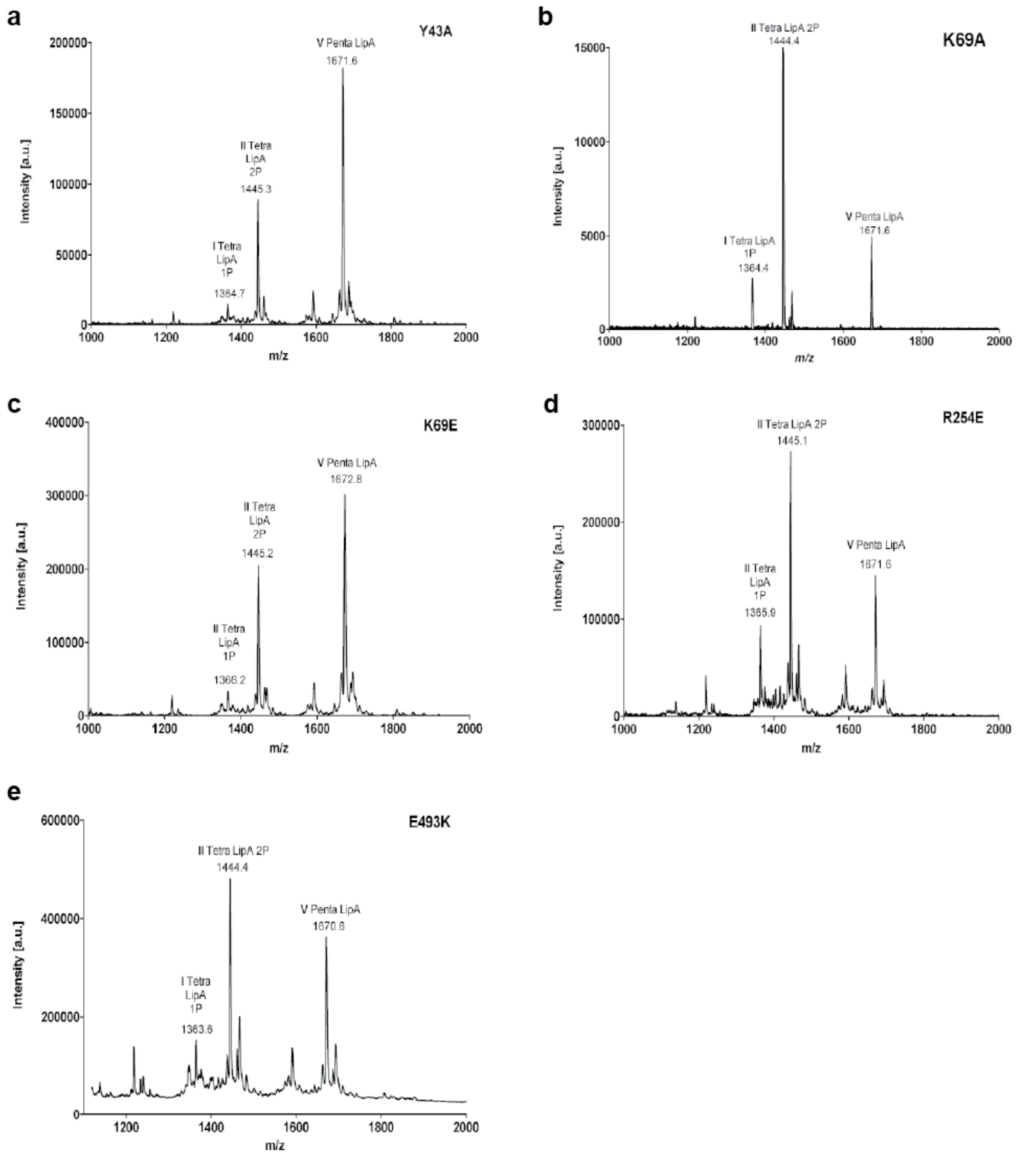

FIGURE S4 | MALDI-TOF spectra of purified lipid A produced by *arnT* mutants that did not present L-Ara4N molecule. The profiles represented were obtained using the negative ion mode. I-II Tetra-acylated lipid A with one or two phosphates molecules. V, Penta-acylated lipid A.

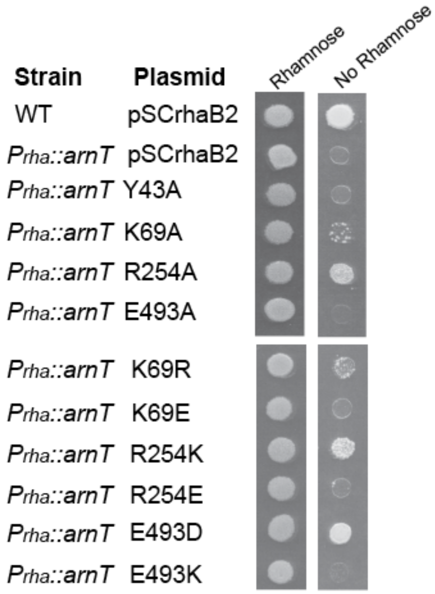

FIGURE S5 | *arnT* mutants cannot rescue growth of *B. cenocepacia* without L-Ara4N synthesis. Plasmids encoding the various ArnT derivatives were introduced into the conditional mutant strain P_{rha}::a*rnT* [1]. Dilutions were spotted in LB plate with or without 0.4% rhamnose.

Supplementary Tables

Table S1 | Strains used in this study.

Strain or Plasmid	Relevant Properties	Source or Reference
Strains		
E. coli		
DH5α	F^- *ϕ*80*lacZ*M15 *endA recA hsdR*($r^-_κ$ $m^-_κ$) *supE thi gyrA relA Δ*(*lacZYA-argF*)*U169*	Laboratory stock
Δ*arnT*	BL21(DE3) Δ*arnT*	[2]
CC118	Δ(*ara leu*) Δ*lac phoA galE galK thi rpsL rpsB argE recA*	Laboratory stock
B. cenocepacia		
K56-2	Parental strain, clinical isolate of the ET12 clone	BCRRC[a] [3]
MH45	K56-2, P_{rha}::a*rnT*	[1]
MH55	Δ*arnT-arnBC*$^+$ *lptG*$_{D31H}$, *lptG* suppressor strain	[1]
Plasmids		
pAH01	pBAD vector expressing Flag-Wzx-K367-PhoA	[4]
pAH18	pBAD vector inducible with arabinose, encoding an N-terminal FLAG and C-terminal for PhoA fusion.	[4]
pAH1809	pBAD vector expressing Flag-Wzx-T242-PhoA	[4]
pBAD24	Expression vector inducible with arabinose, for C-terminal FLAG-10xHis fusions, Ap^R	[5]
pHASoxYZ	*E. coli tatA* promoter controlling expression of *P. panthotrophus* HA-*soxY* and *soxZ* in pSU20, Cm^R	[6]
pMH494	pSCrhaB2-*arnT*$_{Se}$	Hamad and Valvano, *in preparation.*
pRK2013	Helper plasmid used for bacterial conjugation; Km^R	[7]
pSCrhaB2	Expression vector inducible with rhamnose. Broad host range replicative vector; Tp^R	[8]
pFT1	pBAD expressing ArnT-$_{FLAG\text{-}10xHis}$	This study
pFT3	pSCrhaB2, $ArnT_{FLAG\text{-}10xHis}$	This study
pFT4	pAH18, $_{FLAG\text{-}}ArnT_{Bc\text{-}PhoA}$	This study
pFT5	pFT1, $ArnT_{C154A}$	This study
pFT6	pFT1, $ArnT_{C176A}$	This study
pFT7	pFT1, $ArnT_{Cysless}$	This study
pFT12	pFT3, $ArnT_{C154A}$	This study
pFT13	pFT3, $ArnT_{C176A}$	This study
pFT14	pFT3, $ArnT_{Cysless}$	This study
pFT15	pAH18, $_{FLAG\text{-}}ArnT_{Se\text{-}PhoA}$	This study
pFT20	pFT7, $ArnT_{K324C}$	This study
pFT21	pFT7, $ArnT_{D350C}$	This study
pFT28	pFT7, $ArnT_{L385C}$	This study
pFT38	pFT7, $ArnT_{N62C}$	This study

pFT39	pFT7, $ArnT_{G87C}$	This study
pFT40	pFT7, $ArnT_{N239C}$	This study
pFT41	pFT7, $ArnT_{S321C}$	This study
pFT46	pFT7, $ArnT_{R380C}$	This study
pFT47	pFT7, $ArnT_{D439C}$	This study
pFT48	pFT7, $ArnT_{E493C}$	This study
pFT52	pFT7, $ArnT_{G264C}$	This study
pFT54	pBAD, $ArnT_{Se\ (FLAG\text{-}10xHis)}$	This study
pFT55	pFT7, $ArnT_{R254C}$	This study
pFT56	pFT7, $ArnT_{Q530C}$	This study
pFT61	pFT7, $ArnT_{H137C}$	This study
pFT62	pFT7, $ArnT_{I288C}$	This study
pFT63	pFT7, $ArnT_{W209C}$	This study
pFT64	pFT7, $ArnT_{R544C}$	This study
pFT70	pFT54,$ArnT_{Se\text{-}C148A/C149A}$	This study
pFT72	pFT7, $ArnT_{N411C}$	This study
pFT73	pFT7, $ArnT_{W512C}$	This study
pFT76	pFT7, $ArnT_{L35C}$	This study
pFT77	pFT7, $ArnT_{K69C}$	This study
pFT79	pFT7, $ArnT_{F247C}$	This study
pFT80	pFT7, $ArnT_{Q297C}$	This study
pFT86	pFT7, $ArnT_{Y43C}$	This study
pFT87	pFT7, $ArnT_{V106C}$	This study
pFT92	pFT3, $ArnT_{R254A}$	This study
pFT93	pFT3, $ArnT_{E493A}$	This study
pFT94	pFT7, $ArnT_{V332C}$	This study
pFT97	pFT7, $ArnT_{I309C}$	This study
pFT98	pFT7, $ArnT_{T422C}$	This study
pFT109	pFT7, $ArnT_{R550C}$	This study
pFT110	pFT3, $ArnT_{K69A}$	This study
pFT113	pFT7, $ArnT_{L278C}$	This study
pFT116	pFT54, $ArnT_{Se\text{-}R506C}$	This study
pFT117	pFT54, $ArnT_{Se\text{-}Q544C}$	This study
pFT119	pFT7, $ArnT_{H483C}$	This study
pFT120	pFT7, $ArnT_{Y468C}$	This study
pFT121	pFT3, $ArnT_{Y43A}$	This study
pFT130	pFT3, $ArnT_{R254K}$	This study
pFT131	pFT3, $ArnT_{R254E}$	This study
pFT132	pFT3, $ArnT_{E493D}$	This study
pFT133	pFT3, $ArnT_{E493K}$	This study
pFT134	pFT54, $ArnT_{Se\text{-}E438C}$	This study
pFT135	pFT54, $ArnT_{Se\text{-}D469C}$	This study
pFT138	pFT3, $ArnT_{K69R}$	This study
pFT139	pFT3, $ArnT_{K69E}$	This study
pFT143	pFT54, $ArnT_{Se\text{-}P546A}$	This study
pFT144	pFT143, $ArnT_{Se\text{-}Y36A}$	This study
pFT145	pFT143, $ArnT_{Se\text{-}K62A}$	This study
pFT146	pFT143, $ArnT_{Se\text{-}E478A}$	This study
pFT195	pFT7, $ArnT_{R42C\text{-}Y43A}$	This study
pFT196	pFT7, $ArnT_{E682C\text{-}K69A}$	This study
pFT197	pFT7, $ArnT_{R253C\text{-}R254A}$	This study

pFT198	pFT7, $ArnT_{D492C\text{-}E493A}$	This study
pFT210	pFT7, $ArnT_{F138C}$	This study
pFT211	pFT7, $ArnT_{N139C}$	This study
pFT213	pFT7, $ArnT_{R161C}$	This study
pFT214	pFT7, $ArnT_{S186C}$	This study
pFT215	pFT7, $ArnT_{K187C}$	This study
pFT216	pFT7, $ArnT_{H322C}$	This study
pFT217	pFT7, $ArnT_{S323C}$	This study

[a]*B. cenocepacia* complex Research and Referral Repository for Canadian CF Clinics.

Table 2 | Primers used in this study**.**

**Primer**	**DNA Sequence**	**Restriction site**
252	5'-GATTAGCGGATCCTACCTGA	None
258	5'-GACCGCTTCTGCGTTCTGAT	None
6238	5'-GCGCTGTCGCTCgcgTCGCTGCTGCTCGCGCAG	None
6239	5'-CTGCGCGAGCAGCAGCGAcgcGAGCGACAGCGC	None
6240	5'-GGCTGGATGTGGGCGgcgTGGGCCGCGATGGCG	None
6241	5'-CGCCATCGCGGCCCAcgcCGCCCACATCCAGCC	None
6269	5'-aaagaattcATGAACGATACGCCGTCGAGGC	*Eco*RI
6270	5'-aaagtcgactcCGATTGCGGTTTCTCGACGATC	*Sal*I
6385	5'-CAGCGGAACCCCGAGTTCTTCAAC	None
6421	5'-aaagtcgacCGATTGCGGTTTCTCGACGATC	*Sal*I
6475	5'-aaagaattcATGAAATCGATACGCTATTATC	*Eco*RI
6476	5'-aaagtcgactcTTTAGGCCGATACTGAATTAAC	*Sal*I
6716	5'-aaatctagagAACGATACGCCGTCGAGGCTAC	*Xba*I
7178	5'-aaatctagagAAATCGATACGCTATTATCTGGC	*Xba*I
7182	5'- aaagtcgacTTTAGGCCGATACTGAATTAAC	*Sal*I

Restriction sites are underlined.

Supplementary References

1. Hamad, M. A., Di Lorenzo, F., Molinaro, A., Valvano, M. A. Aminoarabinose is essential for lipopolysaccharide export and intrinsic antimicrobial peptide resistance in *Burkholderia cenocepacia*. *Mol Microbiol* **85**, 962-974 (2012).

2. Impellitteri, N. A., Merten, J. A., Bretscher, L. E., Klug, C. S. Identification of a functionally important loop in *Salmonella typhimurium* ArnT. *Biochemistry* **49**, 29-35 (2010).

3. Mahenthiralingam, E*., et al.* Diagnostically and experimentally useful panel of strains from the *Burkholderia cepacia* complex. *J Clin Microbiol* **38**, 910-913 (2000).

4. Marolda, C. L*., et al.* Membrane topology and identification of critical amino acid residues in the Wzx O-antigen translocase from *Escherichia coli* O157:H4. *J Bacteriol* **192**, 6160-6171 (2010).

5. Guzman, L. M., Belin, D., Carson, M. J., Beckwith, J. Tight regulation, modulation, and high-level expression by vectors containing the arabinose PBAD promoter. *J Bacteriol* **177**, 4121-4130 (1995).

6. Bauer, J., Fritsch, M. J., Palmer, T., Unden, G. Topology and accessibility of the transmembrane helices and the sensory site in the bifunctional transporter DcuB of *Escherichia coli*. *Biochemistry* **50**, 5925-5938 (2011).

7. Figurski, D. H., Helinski, D. R. Replication of an origin-containing derivative of plasmid RK2 dependent on a plasmid function provided *in trans*. *Proc Natl Acad Sci U S A* **76**, 1648-1652 (1979).

8. Cardona, S. T., Valvano, M. A. An expression vector containing a rhamnose-inducible promoter provides tightly regulated gene expression in *Burkholderia cenocepacia*. *Plasmid* **54**, 219-228 (2005).
